# Overcoming beta-agonist tolerance: high dose salbutamol and ipratropium bromide. Two randomised controlled trials

**DOI:** 10.1186/1465-9921-8-19

**Published:** 2007-03-06

**Authors:** Sarah Haney, Robert J Hancox

**Affiliations:** 1 Department of Respiratory Medicine, Sunderland Royal Hospital, Sunderland, UK; 2 Department Respiratory Medicine, Waikato Hospital, Hamilton, New Zealand; 3 Dunedin Multidisciplinary Health and Development Research Unit, Dunedin School of Medicine, University of Otago, Dunedin, New Zealand

## Abstract

**Background:**

Asthmatics treated with long-acting beta-agonists have a reduced bronchodilator response to moderate doses of inhaled short acting beta-agonists during acute bronchoconstriction. It is not known if the response to higher doses of nebulised beta-agonists or other bronchodilators is impaired. We assessed the effect of long-acting beta-agonist treatment on the response to 5 mg nebulised salbutamol and to ipratropium bromide.

**Methods:**

Two double-blind, placebo-controlled, crossover studies of inhaled formoterol 12 μg twice daily in patients with asthma.

*High-dose salbutamol*: 36 hours after the last dose of 1 week of formoterol or placebo treatment, 11 subjects inhaled methacholine to produce a 20% fall in FEV_1_. Salbutamol 5 mg was then administered via nebuliser and the FEV_1 _was monitored for 20 minutes. *Ipratropium*: 36 hours after the last dose of 1 week of formoterol or placebo treatment, 11 subjects inhaled 4.5% saline to produce a 20% fall in FEV_1_. Salbutamol 200 μg or ipratropium bromide 40 μg was then inhaled and the FEV_1 _was monitored for 30 minutes. Four study arms compared the response to each bronchodilator after formoterol and placebo. Analyses compared the area under the bronchodilator response curves, adjusting for changes in pre-challenge FEV_1_, dose of provocational agent and FEV_1 _fall during the challenge procedure.

**Results:**

The response to nebulised salbutamol was 15% lower after formoterol therapy compared to placebo (95% confidence 5 to 25%, p = 0.008). The response to ipratropium was unchanged.

**Conclusion:**

Long-acting beta-agonist treatment induces tolerance to the bronchodilator effect of beta-agonists, which is not overcome by higher dose nebulised salbutamol. However, the bronchodilator response to ipratropium bromide is unaffected.

## Background

Patients with asthma who are poorly controlled on inhaled corticosteroids are often prescribed long-acting beta-agonists [1,2]. However, most asthmatics continue to need short acting beta-agonists for relief of breakthrough symptoms and for treatment during asthma exacerbations. Despite accumulating evidence that tolerance develops to the bronchodilator effects of beta-agonists, the effect of regular long-acting beta-agonists on the response to treatment of exacerbations is rarely considered in treatment guidelines.

Tolerance to the systemic [3] and bronchoprotective [4] effects of beta-agonists is known to occur during regular beta-agonist use. It was previously thought that tolerance to bronchodilation did not occur. However, recent studies have shown that tolerance to bronchodilation is easily demonstrated when tested from a bronchoconstricted state, such as after methacholine or exercise challenge [5-13]. Patients using regular long-acting beta-agonists may therefore be less responsive to further beta-agonist treatment during acute bronchoconstriction. This is potentially a serious problem for patients who are taking these drugs and experience an exacerbation of asthma.

Studies of bronchodilator tolerance have demonstrated that regular beta-agonist treatment impairs the bronchodilator response to moderate doses of up to 400 μg of salbutamol [5-12]. It has been suggested that this tolerance can easily be overcome by administering higher doses of beta-agonists, but this has never been formally examined [14]. Treatment of asthma exacerbations in emergency departments usually includes higher doses of beta-agonists given via a nebuliser, typically up to 5 mg salbutamol [1,2]. There have been no randomised controlled trials of the effects of regular long-acting beta-agonists on this emergency treatment.

It is also unknown whether beta-agonist tolerance use affects the airway response to other bronchodilators. The anticholinergic bronchodilator ipratropium bromide is often added to emergency asthma treatment in severe bronchoconstriction and in those who respond poorly to beta-agonist alone [2,15]. It is likely that the bronchodilator response to ipratropium is not affected by prior long-acting beta-agonist treatment since it acts via a different receptor. However, beta_2_-receptors and muscarinic cholinergic receptors share some intracellular secondary messengers and their signalling pathways may interact [16,17]. Hence it is plausible that bronchodilator responsiveness to ipratropium could be affected by beta-agonist tolerance. Moreover, it is possible that beta-agonists have a non-specific effect on airway smooth muscle tone thereby altering the response to other bronchodilators regardless of intracellular signalling pathways [18,19].

Given the role of high dose beta-agonist and ipratropium bromide in the management of asthma exacerbations, it is important to assess their effectiveness in the setting of long-acting beta-agonist treatment. We performed two studies to address the following questions:

Study 1: Does high dose salbutamol overcome the bronchodilator tolerance associated with regular long-acting beta-agonist use?

Study 2: Does beta-agonist bronchodilator tolerance affect the bronchodilator response to ipratropium bromide?

## Methods

### Subjects

Subjects had physician-diagnosed asthma with a provocative dose to cause a 20% fall in FEV_1 _(PD_20_) less than 1.5 mg of methacholine (study 1) or less than 30 ml saline (study 2) at screening (see below). Subjects were not using long-acting beta-agonists prior to the study and had not changed their asthma medications nor had a respiratory tract infection for 6 weeks prior to study entry. The studies were approved by the Waikato Ethics Committee. All subjects provided written informed consent.

### Study design

Both studies were random-order, double-blind, cross-over studies comparing formoterol 12 μg twice daily with placebo. Randomisation was by computer-coded bottles of identical capsules for use with the Foradil Aeroliser (*Novartis, Auckland, New Zealand*). Additional beta-agonists were not allowed throughout the studies. Ipratropium was used for symptom relief.

### Study 1: High-dose salbutamol

This was a crossover study with 2 arms. Subjects inhaled formoterol or placebo twice daily for 1 week. Thirty-six hours after the last dose, subjects underwent a methacholine challenge and salbutamol response as described below. Subjects then crossed to the other medication for a repeat sequence of 1 week of treatment, methacholine challenge and salbutamol response.

Methacholine challenge was performed using a modified Yan technique [20]. Subjects inhaled doubling doses of nebulised methacholine until the FEV_1 _had fallen by ≥ 20%. The PD_20 _methacholine was calculated by linear interpolation of the last two doses. Immediately after completion of the methacholine challenge, subjects were given 5 mg salbutamol via a Sidestream nebuliser and System 22 aerosol mask (*Profile, Bognor Regis, UK*) powered by oxygen at a rate of 6 L/minute. FEV_1 _was measured at 5, 10, 15 and 20 minutes after starting the nebuliser.

### Study 2: Ipratropium bromide

This was a crossover study with four arms. For each arm subjects inhaled formoterol 12 μg or placebo twice daily for 1 week. Thirty-six hours after the last dose, subjects underwent a hypertonic saline challenge and bronchodilator response as described below. Subjects then received the alternative medication and started the next arm of the study. Two study arms compared the bronchodilator response to salbutamol after formoterol and placebo treatment and the other two arms compared the response to ipratropium bromide after formoterol and placebo.

Hypertonic saline challenge was performed according to the method of the European Respiratory Society [21]. Subjects inhaled 4.5% saline until the FEV_1 _fell by ≥ 20%. The PD_20 _saline was calculated by linear interpolation of the last two doses. Immediately after the challenge, 200 μg salbutamol or 40 μg ipratropium was given via a metered dose inhaler and large volume spacer. FEV_1 _was measured 5, 10, 15, 20 and 30 minutes after the bronchodilator.

### Analysis

The main outcome measurement was the area under the salbutamol or ipratropium response curves (AUC), expressed as a percentage of the fall in FEV_1 _induced by challenge. Comparisons between treatments were adjusted for baseline FEV_1 _and dose of provocational agent given using analysis of covariance. A secondary outcome for each study was the final post-bronchodilator FEV_1_, which was also adjusted for the fall in FEV_1 _during challenge. Post hoc comparisons between placebo and formoterol treatments were were made using Tukey's exact method. Comparisons of pre-challenge FEV_1 _and of PD_20 _were made using paired Student's t-tests. PD_20 _values were log-transformed for analysis.

Previous studies indicated that a sample size of 12 subjects would detect a 30% fall in AUC with 80% power at α = 0.05 [5].

## Results

### Study 1: High dose salbutamol

Eleven subjects were recruited and completed the study. Baseline characteristics of the subjects are shown in table 1. There was no significant effect of order of treatment on AUC, pre-challenge FEV_1 _or PD_20 _methacholine. The pre-challenge FEV_1 _and PD_20 _methacholine were not significantly different between placebo and formoterol arms (table 2).

**Table 1:** Baseline characteristics of subjects

Study	1. High dose salbutamol	2. Ipratropium
Number of subjects	11	11
Male	3	3
Age (range)	37 (22 to 62)	34 (19 to 53)
Number on inhaled steroids Dose range (μg)(budesonide equivalent)	9 (200 to 1200)	10 (200 to 2000)
FEV_1_% predicted at screening (range)	79 (61 to 101)	86 (65 to 103)

**Table 2:** Results of the high dose salbutamol study (study 1)

Treatment	Placebo	Formoterol
Pre-challenge FEV_1 _(L) (SD)	2.55 (0.75)	2.57 (0.72)
Post challenge FEV_1 _(L) (SD)	1.92 (0.59)	2.00 (0.51)
PD_20 _methacholine (mg) (geometric mean, 95% CI)	0.08 (0.03, 0.22)	0.10 (0.04, 0.25)

AUC salbutamol (%.time) (SD)	412 (140)	339 (201)
Difference: paired t-test (95% CI)	73 (-4, 149) p = 0.06	
Difference: covariate analysis (placebo-formoterol) (least squares means, 95% CI)	63 (22, 105) p = 0.008	

Post-salbutamol FEV_1 _at 20 mins (L) (SD)	2.79 (0.86)	2.67 (0.84)
Difference: paired t-test (95% CI)	0.12 (0.03, 0.20) p = 0.008	
Difference: covariate analysis (placebo-formoterol) (least squares means, 95% CI)	0.12 (0.06, 0.19) p = 0.003	

**Figure 1:**
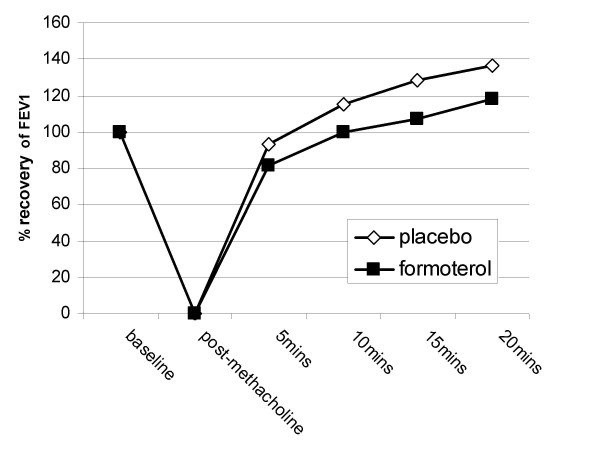
Response to high dose salbutamol (n = 11).

The salbutamol response, expressed as area under the curve, was 15% lower in the formoterol period compared to placebo (95% confidence interval 5 to 25%). This was a significant difference when taking the covariates into account (p = 0.008) (table 2, figure 1).

The final FEV_1 _20 minutes after challenge after 5 mg salbutamol was 2.67 L in the formoterol arm compared to 2.79 L after placebo (95% CI for difference 0.06 to 0.19 L; p = 0.003).

### Study 2: Ipratropium bromide

Thirteen subjects were recruited to the study. Two subjects withdrew during the first treatment period, one with uncontrolled asthma and one with a respiratory infection (taking formoterol and placebo respectively). Baseline characteristics are shown in table 1. There was no effect of order of treatment.

The mean pre-challenge FEV_1 _and PD_20 _saline did not change significantly between formoterol and placebo arms. However, one subject experienced a less than 15% fall in FEV_1 _with inhalation of saline after taking formoterol. For analytical purposes, the PD_20 _saline for these challenges has been assumed to be 30 ml (the maximum value available for inhalation). Exclusion of this subject from analysis does not materially alter the results.

**Figure 2:**
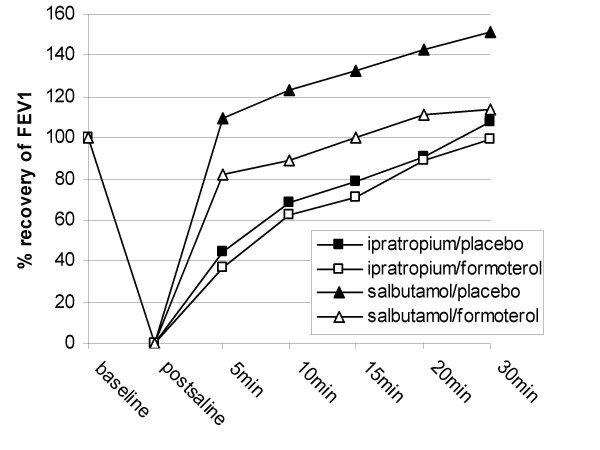
Response to salbutamol and ipratropium bromide (n = 11).

The response to ipratropium, expressed as the AUC, was not significantly different between formoterol or placebo treatment arms (table 3, figure 2). The final FEV_1_, 30 minutes after ipratropium was not significantly different between the formoterol or placebo arms.

**Table 3:** Results of the ipratropium study (study 2)

Bronchodilator	Ipratropium		Salbutamol	
Treatment	Placebo	Formoterol	Placebo	Formoterol

Pre-challenge FEV_1 _(L) (SD)	2.77 (0.50)	2.67 (0.41)	2.62 (0.50)	2.67 (0.63)
Post-challenge FEV_1_(L)(SD)	2.15 (0.38)	2.07 (0.37)	2.06 (0.41)	2.11 (0.60)
PD_20 _saline (ml) (95% CI)	11.8 (7.4–19)	8.0 (4.6–13.8)	8.9 (4.9–16.0)	8.6 (5.0–14.6)

AUC %.time (SD)	447 (215)	401 (154)	759 (203)	557 (131)
Difference: paired t-test (95% CI)	138 (-48,138) p = 0.303		202 (66, 339) p = 0.008	
Difference: covariate analysis (placebo-formoterol) (least squares mean, 95% CI)	88 (-31, 207) p = 0.126		202 (42, 362) p = 0.019	

FEV_1 _at 30 mins (L) (SD)	2.81 (0.54)	2.67 (0.45)	2.91 (0.56)	2.74 (0.64)
Difference: paired t-test (95% CI)	0.22 (0–0.30) p = 0.056		0.10 (-0.05, 0.39) p = 0.117	
Difference: covariate analysis (placebo-formoterol) (least squares mean, 95% CI)	0.11 (-0.06, 0.28) p = 0.172		0.22 (0.05, 0.38) p = 0.015	

The response to salbutamol was reduced in the formoterol arm compared to placebo. The mean difference in the AUC was 26% (95% CI 6 to 46%; p = 0.02) (table 3, figure 2). The FEV_1 _30 minutes after salbutamol was also lower in the formoterol arm compared to placebo, 2.74 L compared to 2.91 L (95% CI for difference 0.05 to 0.38; p = 0.02 by covariate analysis).

## Discussion

These studies confirm that the response to salbutamol is reduced in subjects taking long-acting beta-agonists. This reduction in response was evident even when 5 mg salbutamol was given via nebuliser. By contrast, the bronchodilator response to ipratropium was not affected by prior use of formoterol.

It is known that tolerance to bronchodilation occurs following regular formoterol. This has usually been demonstrated by measuring the bronchodilator response to up to 400 μg of salbutamol via metered dose inhaler. In terms of bronchodilation, 2.5 mg via a nebuliser is equivalent to 800 μg via metered dose inhaler and spacer [22]. We used a 5 mg dose of nebulised salbutamol in this study as this is commonly used in the emergency treatment of asthma. Higher single doses of salbutamol in a nebuliser are not recommended [2]. We found that the response to 5 mg of salbutamol was impaired after 1 week of regular formoterol use, indicating that patients using long-acting beta-agonists are likely to respond poorly to first-line treatment of acute severe asthma even if high doses of nebulised beta-agonist are used. Although the differences in the dose-response curves and final FEV_1 _shown in this study may appear modest, it has been shown that the degree of beta-agonist tolerance increases with the degree of bronchoconstriction [7]. Patients with acute severe asthma will have much more severe bronchoconstriction than the 20% fall in FEV_1 _induced in this study [2,23]. They are therefore likely to have a much greater impairment in response to beta-agonists. In practice, many patients are given repeated doses of beta-agonists via nebuliser during an acute exacerbation. It was not possible to test the effects of repeated nebulisations using this model owing to the time taken for nebulisation (around 5 minutes) and the effect of spontaneous recovery from methacholine-induced bronchoconstriction.

The findings of the salbutamol study appear to contrast with those of a study which found no difference in the response to emergency department beta-agonist treatment between patients who had been taking long-acting beta-agonists and those who had not [24]. However, this emergency department study did not assess the preceding use of short-acting beta-agonists. Since almost all asthmatics take large doses of beta-agonists before presenting to hospital [25] and tolerance to beta-agonists develops rapidly [11], it is highly likely that the 'control' group had also developed tolerance.

The bronchodilator response to ipratropium bromide was not impaired by prior use of long-acting beta-agonist. This confirms that the effect that we have interpreted as beta-agonist tolerance is specific to beta-agonists and unlikely to be due to a non-specific change in airway smooth muscle responsiveness. The intracellular effects of both beta-agonists and cholinergic agents are now known to be far more complex than action on single second messenger systems and there are plausible mechanisms of interaction between them [16,26]. Muscarinic agonists appear to reduce the potency of beta-agonist bronchodilation, possibly through an effect on adenylyl cyclase [17]. The converse effect of muscarinic antagonists has been more difficult to assess [27]. Acetylcholine in human airways acts via muscarinic receptors to decrease cyclic AMP and reduce intracellular calcium release, thereby causing bronchoconstriction. Ipratropium blocks these actions, thereby reducing smooth muscle tone and causing bronchodilation. Cyclic AMP is also a key second-messenger in the response to beta-receptor activation. Regular exposure to beta-agonists appears to uncouple the beta-receptor from cAMP production [28]. Therefore it seemed possible that beta-agonist tolerance would also have an effect on the response to ipratropium. However, this was not discernible in our study. The addition of ipratropium bromide to beta-agonist treatment in acute severe asthma improves bronchodilation [15], whereas it affords little additional benefit in stable asthma [29]. It is possible that this is because the response to beta-agonist in severe asthma is impaired because of prior beta-agonist treatment, whereas the response to anticholinergic treatment is maintained. In this study the bronchodilator response to salbutamol was more rapid and greater than to ipratropium bromide, even after tolerance had developed. However, the effect of beta-agonist tolerance increases with increasing levels of bronchoconstriction [7]. In severe asthma the modest response to ipratropium bromide may become more important.

These studies used formoterol as the long-acting beta-agonist. Studies in the emergency department have focussed on patients taking salmeterol [24]. Salmeterol is a partial agonist at the beta-receptor compared to formoterol and theoretically it may be less likely to induce tolerance. Alternatively, salmeterol could act as a partial antagonist to salbutamol and reduce the effects of salbutamol still further. In practice, trials that have compared bronchodilator tolerance using the challenge-rescue model have not found any difference between salmeterol and formoterol [8,10].

We have interpreted the reduction in response to beta-agonists as tolerance or tachyphylaxis to the effects of beta-agonists. Downregulation of the beta-receptors both in terms of receptor number and intracellular response to receptor stimulation is a pharmacologically predictable effect of repeated receptor stimulation [28]. An alternative explanation for the reduced response to beta-agonist is that some beta-receptors were still occupied by formoterol molecules at the time of the test and were therefore not available to bind to salbutamol, reducing its effect. We measured the bronchodilator response 36 hours after the last dose of formoterol. This is outside the normal duration of action of formoterol [30] and receptor occupancy should be minimal at this time. The 36-hour withdrawal of formoterol also minimises confounding of the results by large changes in pre-challenge FEV_1 _and PD_20_. Although one subject showed a bronchoprotective effect at this time, overall there were no significant differences in the pre-challenge FEV_1 _or PD_20 _values. However, it is also known that beta-receptors recover rapidly following downregulation [31] and the timing of challenges 36 hours after the last dose of long-acting beta-agonist may mean that the tolerance measured in these trials was actually lower than that experienced by patients who continue take their long-acting beta-agonists twice daily [12].

There are potential limitations to our study design. First, this challenge-rescue model does not accurately reflect all of the pathophysiology of acute asthma, where airway inflammation, oedema and mucus production play important roles in airway obstruction. Nevertheless, airway smooth muscle contraction is important and is the main target of emergency bronchodilator treatment. In addition, not all of the subjects in these studies were taking inhaled corticosteroids, whereas current guidelines recommend that long-acting beta-agonists are used in conjunction with inhaled corticosteroids [1,2]. There is conflicting evidence on whether systemic corticosteroids may partly reverse tolerance [32]. However, it is known that stable doses of inhaled corticosteroids do not influence the development of tolerance [5] and hence the use of inhaled corticosteroids was not a requirement in our selection of subjects.

This study is the first to demonstrate that beta-agonist bronchodilator tolerance can be demonstrated using hypertonic saline. Most previous studies using the 'challenge-rescue' technique have used methacholine as the challenge agent [5-8,10-12,33]. This would have been inappropriate prior to studying the response to the anticholinergic drug ipratropium. Our findings confirm that the challenge-rescue model for studying bronchodilator tolerance works with both direct and indirect challenges. The bronchial response to hypertonic saline challenge is closely correlated with the response to exercise challenge [34]. A suboptimal response to salbutamol after exercise challenge has also been found when patients use regular beta-agonists [9].

## Conclusion

In summary, these studies confirm that beta-agonist bronchodilator tolerance develops during long-acting beta-agonist treatment. This is not overcome by 5 mg of nebulised salbutamol. The effect is specific to beta-agonists and does not affect the response to ipratropium bromide. Patients using long-acting beta-agonists may respond less well to emergency beta-agonist treatment. Additional treatment with an alternative bronchodilator should be considered early in the course of an exacerbation.

## Abbreviations

AMP – adenosine 5'monophosphate

AUC – area under the curve

FEV_1 _– forced expiratory volume in one second

PD_20 _– dose of bronchoprovocational challenge agent required to produce a fall in FEV_1 _of 20% from baseline

## Competing interests

The author(s) declare that they have no competing interests.

## Authors' contributions

SH contributed to the trial design, acquired the data, performed the statistical analysis and helped to draft the manuscript. RH conceived the study, participated in its design, interpretation of results and helped to draft the manuscript.
